# Ecosystem-Based Adaptation Projects, More than just Adaptation: Analysis of Social Benefits and Costs in Colombia

**DOI:** 10.3390/ijerph16214248

**Published:** 2019-11-01

**Authors:** Carmen Richerzhagen, Jean Carlo Rodríguez de Francisco, Felix Weinsheimer, Alessandro Döhnert, Lukas Kleiner, Marjam Mayer, Julia Morawietz, Eric Philipp

**Affiliations:** German Development Institute/Deutsches Institut für Entwicklungspolitik (DIE), Tulpenfeld 6, 53113 Bonn, Germany; felixweinsheimer@gmx.de (F.W.); alessandrodoehnert@gmail.com (A.D.); lukas.kleiner@web.de (L.K.); Marjam.Mayer@ptb.de (M.M.); juliamorawietz@posteo.de (J.M.); eric.philipp89@gmail.com (E.P.)

**Keywords:** ecosystem-based adaptation, social benefits, climate change, Colombia, water, mangroves

## Abstract

Ecosystem-based Adaptation (EbA) projects are increasingly flourishing throughout the globe on the grounds that EbA constitutes a particularly community-friendly solution for adaptation to climate change as it brings about an array of co-benefits. However, the promotion of EbA projects, by development agencies and conservation NGOs, remains blurry as it has not yet been contrasted against evidence on its effectiveness in delivering these benefits. Employing a political ecology perspective, the applied conceptual framework allows for the assessment of the social benefits and costs that EbA projects generate or reinforce and factors that influence the distribution of these social benefits or costs. This research is done in regards to two EbA projects in Colombia: one in the Andes focusing on water provision services from páramos, and the other in a coastal mangrove focusing on regulation services of extreme coastal events. Based on data collected by a qualitative multi-method approach, we find evidence that the assessed EbA projects generate a wide range of perceived social benefits and costs for the local communities living in the vicinity of the project sites. Furthermore, we identify agent-level (i.e., capitals and preferences) as well as structural factors (communication, participation, local and institutional context) that influence the generation and distribution of those social benefits and costs. Finally, this paper illustrates some of the contradictions and tensions in which EbA projects are implemented and how they may end up affecting the adaptive capacity of the communities involved in EbA projects.

## 1. Introduction

Climate change affects ecosystems and therefore the natural and human populations depending on the services that ecosystems provide [1]. These adverse impacts manifest in different areas of the world and population sectors in various ways: droughts, flooding, pest outbreaks, food stresses and heat waves [2]. According to the IMF [3], climate change bears the largest negative impacts on tropical countries. As extreme poverty affects large parts of the populations in nearly all low-income and many medium-income countries situated in the tropics, climate change exacerbates the vulnerabilities of the already marginalized [4,5]. Ecosystems and their services play a crucial role in adapting to the negative effects of climate change in the form of nature-based solutions.

Ecosystem Services (ES), in the form of provisioning, regulating, supporting, and cultural services, are defined as “benefits people obtain from ecosystems” [6] and understood as contributing to human well-being. Climate change affects ecosystems by interfering with their functioning, impairing the provision of ecosystem services and impacting the vulnerability of social-ecological systems. Ecosystem-based Adaptation (EbA) may be a tool to adapt to and mitigate the adverse effects of climate change on social-ecological systems [7].

EbA has gained significant momentum in the fields of environmental and development policies [8]. There are many EbA projects being implemented in the Global South [9,10] but also in the Global North [11,12]. By definition, EbA is “the use of biodiversity and ecosystem services as part of an overall adaptation strategy to help people to adapt to the adverse effects of climate change” and takes into account “the multiple social, economic and cultural co-benefits for local communities” [13].

Despite the strong promotion of the approach, building evidence of its environmental, social and economic benefits in practice remains work in progress [14]. Emerton [15] stated that there is a lack of demonstrable evidence of EbA approaches in delivering ecosystem service co-benefits—more specifically the aforementioned social co-benefits—that are claimed for them. Besides sparse research on EbA benefits, potential social costs generated by EbA projects remain unknown to many implementing agencies. Another shortcoming on EbA research relates to how benefits and costs are distributed among individuals and social groups at different scales.

Seeking to address this research gap, we engage in qualitative research focusing on two EbA projects in Colombia. We seek to unravel the social benefits and costs that the projects generate and to identify factors that impact the distribution of these benefits and costs. The first project was implemented in a rural setting in the Colombian Andes. The objective was to adapt the watershed ecosystem services to the impacts of climate change, focusing particularly on the provision of fresh water from the Chingaza páramo to local communities and the city of Bogota. Páramo ecosystems extend between the Andean forests upper-line and the perennial snow-border (3200–5000 m above sea level) in the northern Andes. Páramos are not only essential to the provision of watershed ecosystem services but also for carbon sequestration services [16]. The second project under implementation during our field visit was in a peri-urban setting in the southeastern part of the Ciénaga de la Virgen (Ciénaga), a mangrove forest in the city of Cartagena de Indias on the Caribbean coast. Here, the focus was to protect/restore mangroves, canal and improve solid waste management to adapt to extreme weather events and to increase green areas against heat waves. 

The following section presents our novel conceptual framework. Employing a political ecology perspective, we seek to encapsulate the relation between EbA projects, the social benefits and costs these projects generate and their distribution. Section 3 presents the two case studies and the methods applied in our empirical research. Section 4 presents our results and is followed by a discussion. Section 5 provides our concluding remarks.

## 2. Conceptual Framework

We attempt to analyze the social benefits and costs of EbA projects and the factors that impact their distribution based on a conceptual framework inspired by the work of Fisher et al., Daw et al., Lakerveld et al. [17,18,19]. Following on the political ecology vein, we engage in a disaggregated analysis of benefits and costs that accrue from EbA projects and pay particular attention to their distribution among individuals. The measured impacts are qualified on the basis of the perception of community members living in the vicinity of the analyzed EbA projects. In this context, it is important to recognize the multiple forms of access and control over resources and their implications for environmental health and sustainable livelihoods [20]. In contrast to Fisher et al. [17], the starting point of our analysis is the particular EbA measure and not the ES. 

**Figure 1:**
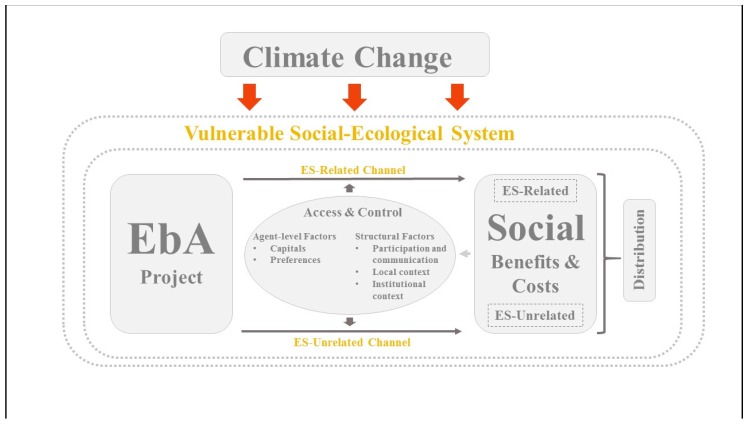
The conceptual framework.

We argue that EbA projects can generate social impacts (benefits and costs) through two channels. In the first case (channel), benefits are generated through the direct use of ecosystem services. In the second case (channel), benefits are generated through the EbA project but are not related to ecosystem services. We name the first channel ES-related channel (see Figure 1). Benefits that refer to the ES-related channel are the restoration of maintenance and enhancement of ecosystems (e.g., mangroves) aiming to ensure the provision of ES. ES-related costs can be, e.g., the loss of productive land. EbA projects can also generate benefits and costs that are not directly related to ES and that can be linked to overarching procedural and participatory elements of the project. These are captured in the ES-unrelated channel (see Figure 1). Examples for benefits include, e.g., increased influence and participation of women in decision-making processes [21] and increased capacity and knowledge of people that may enable them to establish resilience strategies [15]. EbA projects may create social costs that are ES-unrelated, e.g., by increasing an already disproportionate work burden on women (on the field of water-supply) [22].

We contend that “access and control form the social and political dynamic through which people interact around ecosystem services” [17]. Control refers to “social processes governing access” [17,23]. Fisher et al. [17] argue that, especially from the perspective of poor and vulnerable members of society, access and control to ES are often more important than their aggregated availability. In fact, ES-literature suggests a number of characteristics that differentiate people and shape their ability to access ES. For our conceptual framework, we have singled out decisive agent-level characteristics that are likely to influence the distribution of social benefits and costs of EbA projects: on the one hand, capitals, touching upon endowments and entitlements and, on the other hand, preferences, shaping people’s opinion about EbA projects and therefore determining the degree of their individual participation in such projects [17,24,25].

While these characteristics represent agent-level factors, a disaggregated analysis of determinants of EbA-related social benefits and costs also requires the incorporation of certain frame conditions. These include structural factors related to participation, communication, as well as the local and institutional context. Participation and communication determine how the participation of relevant actors is organized and how information is disseminated. The institutional context represents the political environment in which an EbA project is embedded. It considers the capacities, effectiveness, efficiency and commitment of institutional actors such as governmental actors, decision-makers and local authorities, as well as their approach to environmental law enforcement and resource governance. Under local context, we conceive the socio-economic circumstances under which an EbA project is being implemented. It captures elements such as existing practices and networks, social disparities as well as the historic and cultural background.

The analysis of structure–agency characteristics allows combining both “the focus on variations in individual asset ownership and preferences, and the focus on social access and structure” [19]. However, it is important to note that agent-level and structural factors are highly context-specific. They depend on multiple variables (type of EbA project, type of ecosystem, and vulnerability of the project region) and should therefore not be seen as static and structurally given, but rather as dynamic and dialectical. 

The presented conceptual framework allows for the investigation of the generation and distribution of social benefits and costs in ongoing as well as in finalized projects. Indeed, the obtained social benefits and costs may alter the agent-level and structural characteristics that determine people’s access and the distribution of ES through both channels. 

## 3. Research Methods and Cases

### 3.1. Methods

From February to April 2018, we embarked on a field trip to Colombia, applying a qualitative multi-method approach along two case studies. Our data are based on semi-structured individual and group interviews as well as background talks with actors at different levels (government representatives, project implementers and local community members). Additionally, we conducted focus groups with local community members and resorted to participant observation at the project sites. Fieldwork was mainly conducted in Spanish.

Our interviews had different objectives depending on the respective role of the interviewee. Interviews with political and administrative staff at national and local level as well as with project implementers focused on their particular field of expertise [26], and provided us with insights into EbA strategies in Colombia and the practitioners’ evaluation thereof. Interviews with members of the local communities gave us information on their subjective perceptions and individual impressions concerning the implementation of the specific EbA project with a focus on the perceived social benefits and costs and their respective distribution. 

Based on our conceptual framework and bearing in mind the interviewee-specific research objectives, we developed three interview guidelines based on Reid et al. [27]. Our interview guidelines allowed us to work in a structured and open way [28] and left room for asking interviewees for further elaborations or additional information based on their answers. We asked interviewees for examples about adaptation and climate change policy and action in Colombia (policy makers), the individual perception of nature and environmental changes (local level), the EbA decision-making process and project design and implementation (implementers), participation opportunities as well as distributional aspects and social differentiation (all levels).

For the Chingaza case study, we conducted 40 interviews both in Bogotá and on site in the municipalities of La Calera and Choachí. In total, we talked to 71 people who were involved in the “World Bank’s Integrated National Adaptation Project” (INAP) in different ways. The vast majority of our interview partners, namely 65, were members of the communities that participated in the project. Furthermore, we talked to representatives of the implementing organizations—Conservation International (CI) and the Colombian Meteorological, Hydrological and Environmental Studies Institute (IDEAM)—and members of neighboring communities that did not participate in the project.

For the Cartagena case study, we conducted 27 interviews with 46 people, both in Bogotá and on site in Cartagena. Twenty-three of our interview partners were members of Cartagena’s Urban District 6 (Unidad Comunera de Gobierno 6, UCG6). We interviewed further stakeholders including implementers of the Deutsche Gesellschaft für Internationale Zusammenarbeit (GIZ), representatives of national and local governmental project partners and other actors working in the Cienaga de La Virgen. Besides formal and informal interviews, we carried out field visits to the neighborhoods of UCG6 (El Pozón, Nuevo Paraíso, Fredonia, and Olaya Herrera).

Furthermore, we gathered background information on the environmental policy landscape and EbA in Colombia through seven interviews with policy makers, implementers and researchers.

We analyzed our data with inspiration from Philipp Mayrings’ qualitative content analysis [29]. Data analysis followed our conceptual framework; we developed categories for social benefits, social costs, and the factors that determine their generation and distribution. Along these categories, we coded our data, using the software ATLAS.ti. Before applying codes, we grouped the memory protocols and transcripts into different document families. We distinguished between implementers, project partners (e.g., at government level), community members (in the case of Chingaza distinguishing further between different neighborhoods and in the case of Cartagena between the local environmental organizations), and other persons unrelated or somehow related to the two EbA projects (in the case of Cartagena, there are several institutions collaborating with GIZ).

A very general limitation in our research was the fact that interviewees can always have particular interests and biases that may stem from their institutional affiliation or social standing. Due to the circumstances, our interviews sometimes took place under suboptimal conditions, which did not allow for confidentiality. Besides interviewees’ biases, the continuous reflection of our position as researchers was also crucial to become aware of own biases, which could otherwise have created the perception of a sort of social desirability of answers on the part of the interviewees [30]. Security constraints in the neighborhoods of Cartagena required us to walk in the presence of the leaders of local organizations. This considerably limited the degree to which we could select the interviewees ourselves. 

### 3.2. Chingaza Case Study

**Figure 2:**
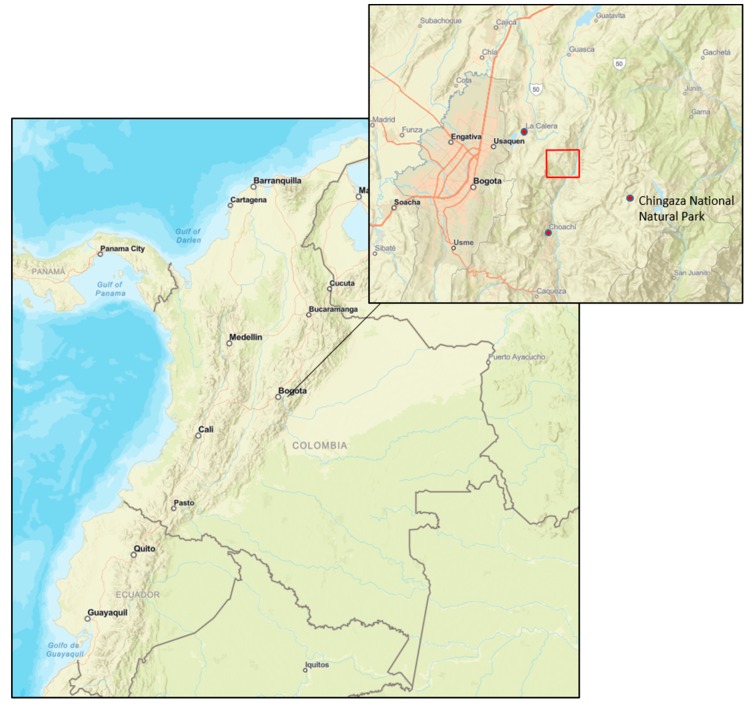
Location of field sites (in red) in Chingaza, Colombia (Adapted from) [33] (mapa digital de Colombia).

The Chingaza páramo (110,000 ha) is located in the eastern Cordillera of the Andes—east of Colombia’s capital, Bogota (see Figure 2). Colombia’s second largest páramo provides 70% of Bogota’s drinking water and caters to nearby municipalities. In Bogotá, nearly 80% of all water originates from the Chingaza Massif [16], more specifically from the Río Blanco watershed that covers approximatively 30% of Chingaza’s surface [31]. Chingaza comprises eleven municipalities in the departments of Cundinamarca and Meta. The importance of the Chingaza páramo in terms of its watershed ecosystem services and its rich flora and fauna led to its declaration as National Natural Park in 1977 (around 44% of the whole páramo) and to the creation of a protective forest reserve (covering 24% of the whole páramo) [32].

Our case study area is located in the buffer area of the Chingaza National Natural Park, between the municipalities of La Calera and Choachí, where the Río Blanco watershed runs. The pattern of land ownership in Chingaza reflects the inequalities of the whole country (landholding classification according to van der Hammen et al. (2015): Big (>200 ha), medium (20–200 ha), small (10–20 ha), micro (3–10 ha) and mini (<3 ha) landholders.). Big and medium landholders (1% and 9%, respectively) control 60% (16% and 44%, respectively) of the whole land. In contrast, micro (63%), mini (21%) and small (7%) landholders control 40% (11%, 16% and 13%, respectively) of the land [32]. 

Our research analyzed one component of the World Bank’s Integrated National Adaptation Project (INAP), which was implemented from 2006 to 2011 by the Colombian Meteorological, Hydrological and Environmental Studies Institute (IDEAM) and the international NGO Conservation International (CI). The project aimed at generating further information on the impacts on climate change, defining policy options and implementing pilot adaptation measures in the most vulnerable areas of Colombia [34]. Crucial to the implementers was to maintain Chingaza’s importance in terms of water and hydropower provision. 

The project focused mostly on micro, mini and small landholders and included activities in two major areas, namely restoration and conservation of native species, as well as adaptation of productive agricultural systems [35]. The assumption behind the project was that climate change will have disastrous consequences for agriculture and will put water for agricultural and human use at risk. 

The project component on restoration and conservation of native species implemented several measures, based on an analysis of the link between land cover and land use on the one hand and hydrological regulation on the other hand [35]. The implementers involved the communities of several veredas (i.e., El Manzano, La Jangada Baja, La Jangada Alta, Mundo Nuevo, El Cerro y La Hoya, La Caja and El Rosario) through hiring community technicians, educational workshops and knowledge exchanges in order to jointly define the most important plants and traditional land use systems. To guarantee people’s commitment in the activities, they implemented signed restoration agreements [16] (Implementer Chingaza, personal communication, 13 February 2018). The communities and the project’s technicians were then responsible for restoration processes of strategically important zones for hydrological regulation such as water springs and riparian buffer zones as well as for the establishment of living fences [35]. 

The project component for adaptation of productive agricultural systems included activities such as the formulation of adaption plans for farm management which were developed by the farmers themselves [16]. Beyond that, the project included actions such as the implementation of organic vegetable gardens, cattle sheds, chicken houses, small-scale irrigation infrastructure and promoted the use of organic fertilizers and making and use of medicinal plants [35]. All those measures were accompanied by capacity-building measures in topics such as sustainable agricultural practices, water saving and use, processing of dairy products or agricultural entrepreneurship [34].

### 3.3. Cartagena Case Study

Cartagena de Indias (Cartagena), capital of the Bolívar Department and located on the shores of the Caribbean Sea, is home to 1,013,389 inhabitants [36]. In economic terms, Cartagena is among the three most important cities in Colombia. In 1984, Cartagena was declared a Touristic, Historical, and Cultural District by UNESCO [37], attracting thousands of national and international tourists every year. Due to this fact, Cartagena is a hotspot for real estate markets, with a booming construction sector for tourism and housing. 

Despite its economic prosperity, the city is characterized by great inequalities. Outside Cartagena’s old city and main touristic monuments, the poverty is very clear: almost 30% of the inhabitants of Cartagena live in poverty and 5.5% in extreme poverty. According to climate models, a 2 °C rise in temperature would lead to a further increase in sea level that would leave 25% of the population and residential properties in the city affected by flooding during high tides. The lowest-income neighborhoods, including those located around the Ciénaga de La Virgen, are most vulnerable to increased flooding [38]. 

**Figure 3:**
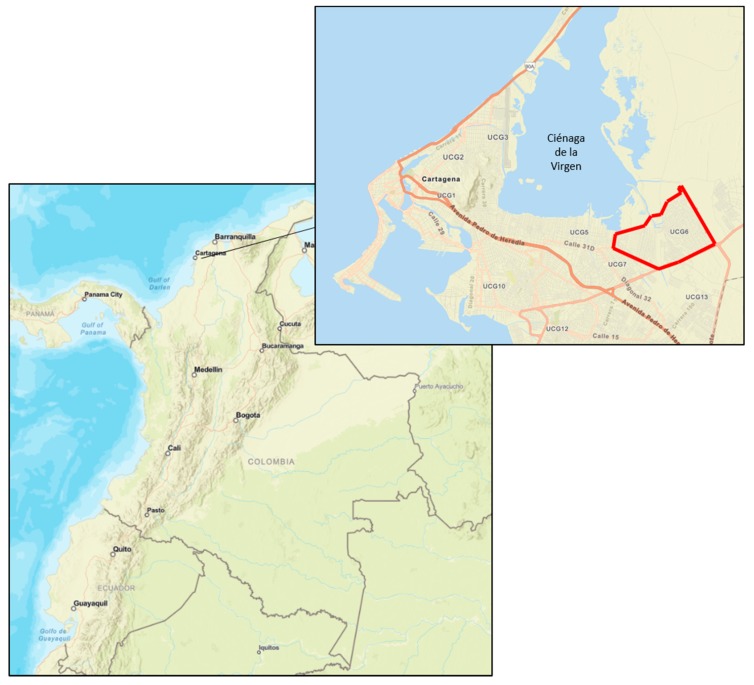
Location of field sites (in red) in Cartagena, Colombia (Adapted from) [33] (mapa digital de Colombia).

Our case study is located in the Unidad Comunera de Gobierno 6 (UCG6), neighboring the southeast stretch of the Ciénaga, more precisely on Olaya Herrera, Fredonia, Nuevo Paraíso and El Pozón neighborhoods (see Figure 3). Here, GIZ’s EbA project “Strategies for ecosystem-based adaptation to climate change in Colombia and Ecuador” is being implemented. The program was started in 2014 and ran until 2018. The program’s main objective was to integrate the EbA approach into relevant policies and planning instruments of national and local authorities as well as to reduce the vulnerability of communities in coastal regions of Colombia and Ecuador to the impacts of climate change [39]. The main measures implemented in Cartagena with respect to the reduction of vulnerability include the recovery and maintenance of canals in an urban area, the conservation and restoration of nearby mangroves forests, and capacity strengthening of local environmental organizations.

The area targeted by GIZ’s EbA project is dominated by residential housing [40,41]. However, some parts of the neighborhoods on the shores of the Ciénaga de la Virgen are still covered by dense mangroves. Between residential settlements and these stretches of mangroves, there are further areas of fragmented forests, bushes and grasslands. Some areas are covered by degraded land. Furthermore, two canals cross the UCG6 (Canal Calicanto Nuevo and Canal Fredonia). Sánchez et al. [42] explained how, for many years, the Ciénaga has been the main receptor of the city’s wastewater and solid waste. Construction debris and solid waste are used to reclaim land from the Cienaga de la Virgen, causing the decrease of its water mirror and mangroves. Land reclamation from the mangrove is sponsored both by low-income residents seeking to obtain housing spaces and by influential sectors that aspire to expand construction areas on sites that offer high-value landscaping services. The neighborhoods in the UCG6 are classified as highly vulnerable to the adverse effects of climate change. Extreme weather events such as heavy rainfall and flooding put pressure on local ecosystems and have devastating impacts on the local communities. These pressures are increased by a lack of environmental awareness and invasive settlement from displaced communities into the Ciénaga. Compared to the other areas of Cartagena, authorities register the highest crime rates [36]. The access to public services also reflects inequalities: 80% of the population do not have access to water supply and 82% do not have access to sewage systems [43]. Over the past decades, invasive settlement in the surrounding of the Ciénaga has been a central part of Cartagena’s urban development. Solid waste management remains a challenge in the area and many municipal contracts to manage and collect solid waste from residential areas and channel wastewater streams through the canals into the Ciénaga are now under investigation for corruption [44].

## 4. Results

### 4.1. Chingaza

#### 4.1.1. Social Benefits and Costs

**Figure 4:**
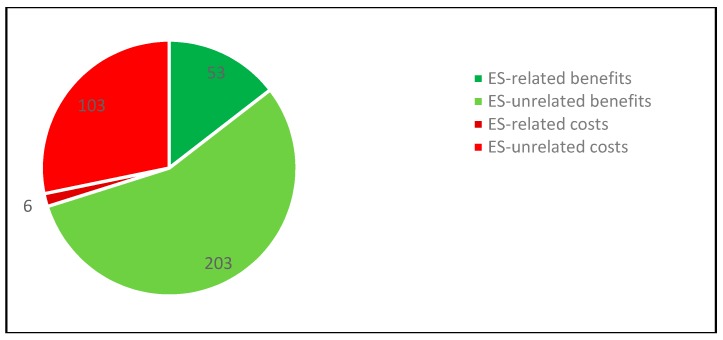
Overall frequency of references relating to social benefits and costs in the Chingaza case study (own representation).

Overall, the frequency of the references made by interviewees suggests that in their perception, social benefits predominate over social costs, ES-unrelated benefits predominate ES-related benefits and ES-unrelated costs predominate ES-related costs (see Figure 4).

##### ES-Related Social Benefits

**Figure 5:**
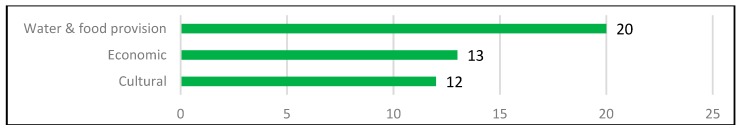
References to ES-related social benefits in the Chingaza case study (own representation).

Community members highlighted INAP’s impact on the provision of water and food (see Figure 5). They especially pointed to increased food security through improved productive agricultural systems (e.g., vegetable gardens and greenhouses) and the project’s positive impact on water quality (Resident Chingaza, personal communication, 15 February 2018). They stressed that through trainings and the provision of inputs (e.g., plants, tools, and greenhouses) community members benefit economically (Residents Chingaza, personal communication, 16 February 2018). New income sources could be opened through the marketing of newly produced goods, e.g., fruit, vegetables and essential oils from an installed production site (Resident Chingaza, personal communication, 15 February 2018).

At the same time, community members stated that INAP disclosed cultural values of ES. For instance, many people mentioned how the project improved the scenic beauty. In this respect, INAP promoted the rootedness of local people in a nature-based peasant culture. As a participant of the focus group put it: “We appreciate that we are farmers. […] We love what we do. […] People started to value their lives and surroundings” (Resident Chingaza, personal communication, 18 February 2018).

##### ES-Unrelated Social Benefits

**Figure 6:**
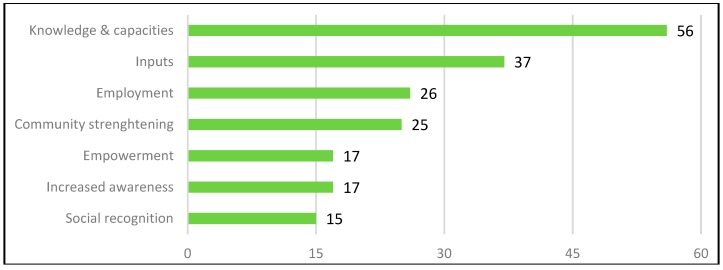
References to ES-unrelated social benefits in the Chingaza case study (own representation).

Our interview partners highly emphasized increased knowledge and capacities—“on institutional level but also knowledge in the people” (Implementer Chingaza, personal communication, 21 February 2018) (see Figure 6). These were conveyed in the course of workshops, meetings and exchanges of experiences, but also by learning on the job. In this context, respondents highlighted that since INAP’s implementation, younger generations have more knowledge about nature and its services: “If I now go to the school, the children talk about water, about climate change” (Resident Chingaza, personal communication, 15 February 2018). These increased capacities naturally manifest in increased awareness of climate change and its impact on local livelihoods. As one community member explains: “[INAP] has opened the eyes of many people and taught them how important it is to conserve the nature and that this also benefits their productivity” (Resident Chingaza, personal communication, 15 February 2018). This has at times led to a behavioral change in that people have stopped logging and burning trees, keep their cattle in a safe distance from the páramo mountains and have started to take better care of their land.

Furthermore, the interview partners identified the inputs provided by the project as benefits. However, they noted that while certain inputs—such as stables or green houses—were beneficial only for the direct recipients, others—e.g., the provision of plants for living fences or riparian buffer zones—generated benefits for the entire community (Resident Chingaza, personal communication, 16 February 2018).

Employment opportunities were another major ES-unrelated benefit for the local communities. The jobs for technicians and auxiliaries were distributed equally across genders and benefitted unskilled and young community members in particular. Certain female respondents highlighted that being given a job and thus contributing to their community’s development made them feel proud of themselves and increased their self-esteem. One respondent added: “through the project I learnt how to voice my thoughts, my concerns and not to keep them locked up inside me” (Resident Chingaza, personal communication, 18 February 2018). 

Besides these positive effects on individual empowerment—especially of women—INAP also helped to support peasant economies and to make farmers more independent from markets (e.g., production of own organic fertilizers). By incorporating local knowledge and relying on the social networks of the technical and auxiliary staff, INAP also led to increased social recognition and perceived appreciation of those that participated in its activities (Implementer Chingaza, personal communication, 21 February 2018). 

Importantly, the project also contributed to community strengthening by facilitating and encouraging the creation of local associations. By fostering cross-community cooperation and ties, the project also helped bringing the different communities in the region closer together. The project helped to overcome tensions that had strained their relations in the past (Resident Chingaza, personal communication, 15 February 2018).

##### ES-Related and ES-Unrelated Social Costs 

**Figure 7:**
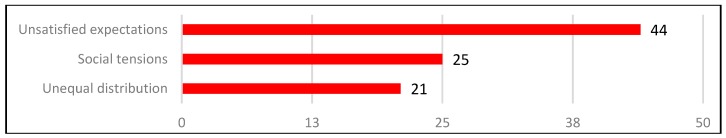
References to ES-unrelated social costs in the Chingaza case study (own representation).

Amongst the most prominent ES-unrelated social costs appearing in the context of INAP respondents alluded to unsatisfied expectations since some people felt that certain “promises were not kept” (Resident Chingaza, personal communication, 15 February 2018) (see Figure 7). At the same time, some respondents were disappointed that INAP did not ensure the continuity of activities and thus “lots of knowledge got lost” (Resident Chingaza, personal communication, 15 February 2018). These perceptions also result from the impression that projects following INAP (e.g., the current páramo project implemented by Bogotá’s water utility) did not hire community assistants resorting to their skills and knowledge, but rather to outside people.

The perceived unequal distribution of jobs and physical inputs as well as opportunities to participate within and across communities provided a further source of discontent (Residents Chingaza, personal communication, 16 February 2018). The project was implemented in an area with high levels of mistrust towards local authorities, governmental entities and implementing bodies (such as the World Bank). In some cases, the mistrust towards these authorities amplified the social tensions between community members (Resident Chingaza, personal communication, 15 February 2018).

Furthermore, perceived social disregard (e.g., in consequence of non-consideration of local needs) and opportunity costs (e.g., land devoted to restoration measures) were mentioned as further social costs.

Only a few ES-related social costs were mentioned by our interview partners (e.g., increased property taxes as a consequence of improved farms or the introduction of non-native species that were perceived as being too water intensive).

#### 4.1.2. Decisive Factors

##### Agent-Level Factors

The consideration of social, human and physical capital played an important role in the generation and distribution of benefits and costs. While INAP was running, it provided jobs to community members in Chingaza, some of which did not require a professional or technical background in the field of adaptation to climate change. Instead, during the recruitment process of project assistants, special attention was given to applicants’ social capital (e.g., their social network and their ability to reach out to and connect different community actors). This enabled especially many women to be hired as project assistants, “[…] [They] got employment and thus were empowered; you could even say they were more and more conquering public spaces” (Implementer Chingaza, personal communication, 22 February 2018). Existing networks in the local communities facilitated the implementation of INAP, as “environmental topics were not a new thing for the community” (Implementer Chingaza, personal communication, 22 February 2018). However, this was mostly done at school, and not with the adult community members. The selection and employment of local community members with strong human capital and technical skills (e.g., degree in forest management) simplified and accelerated project implementation (Resident Chingaza, personal communication, 18 February 2018). In addition, physical capital constituted a key factor that determined both the prerequisites to participate and the selection of implementation sites for project measures. Often, only community members that met specific requirements (e.g., land owners, land with water sources, etc.) were considered as participants of certain project measures: “Project staff came to my house […] because I have a water spring and they wanted to build the greenhouse on my property” (Resident Chingaza, personal communication, 16 February 2018).

Individual preferences influenced the degree of participation in the project and their commitment for the project’s objectives. For example, initially some people preferred not to lend their land properties for the implementation of any restoration or protection measure around water sources, due to the fear of land expropriation. However, after the project advanced, many people where directly contacting project implementers to offer their land for measure’s implementation. “In theory, everyone could participate, but participation depends on individual willingness. However, when people saw that participants were benefitting from the project, they also wanted to participate” (Implementer Chingaza, personal communication, 8 February 2018). 

##### Structural Factors

Participation through inclusive decision-making and engagement is key for the generation and distribution of social benefits. INAP employed a participatory approach that included the communities in the decision-making processes. This resulted in “[…] less conflict occurring [because] the communities decided collectively who were the most vulnerable people who eventually would receive the benefits” (Implementer Chingaza, personal communication, 22 February 2018). This facilitated the generation of social benefits and led to a distribution that was later on perceived as relatively fair.

Communication (what and how) of the implementing organization is crucial for securing people’s participation in and acceptance of a project and can influence individuals’ perceptions of social benefits and costs. The implementing organization considered the local needs of the communities in the Río Blanco watershed: “They incorporated local needs by asking who requires what during the meetings. The community discussed who needed what and decisions were taken jointly” (Residents Chingaza, personal communication, 19 February 2018).

With regard to the institutional context, stakeholders highlighted the role of local authorities. Both implementing organizations and community members noted a lack of engagement on the part of the local authorities in the process of INAP. Although the project team tried to align some of its activities to the overarching development policies of the local government, the regional mayor’s office did not engage in INAP’s activities (Researcher Chingaza, personal communication, 20 February 2018).

With regard to the local context, INAP additionally made use of local knowledge by organizing meetings where the exchange of knowledge between implementers and members of the communities was promoted so that “[…] the community was able to participate in the implementation of measures” (Famer Chingaza, personal communication, 15 February 2018). Local knowledge was included in restoration activities, with respect to planting and using local plant species. However, it was only considered during project implementation. In the earlier planning phase, implementers rather relied on scientific studies.

In the case of Chingaza, the cultural background played an important role for the design of INAP and the selection of the implementation site: “The project is rooted in the Muisca culture, which places a very high value in water and water provision. Therefore, the project was conceived to rescue this indigenous culture” (Implementer Chingaza, personal communication, 20 February 2018). Against the background of this cultural heritage, members of local communities may have been more aware of pressures on water quality and availability, and therefore may have perceived more benefits relating to INAP’s measures geared towards improving this situation.

People’s attitudes and their skepticism regarding outsiders’ interests in securing water resources in Chingaza can be traced back to a perceived history of injustice. In the 1970s, Bogotá’s water utility had worked towards restricting agricultural use of land in the Chingaza region. This had a ruinous impact on farmers’ activities, which still prevails and shapes local communities’ perception of outsiders’ interventions in the water sector (Resident Chingaza, personal communication, 12 February 2018). This history of injustice and skepticism may have hampered and influenced the perception of social benefits and costs.

### 4.2. Cartagena

#### 4.2.1. Social Benefits and Costs

**Figure 8:**
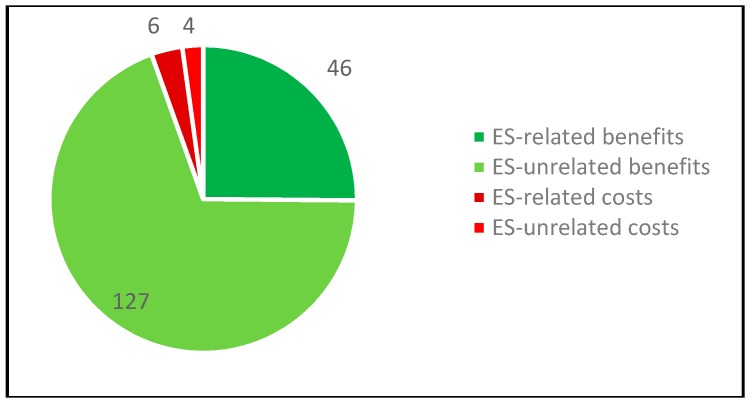
Overall frequency of references relating to social benefits and costs in the Cartagena case study (own representation).

Overall, the frequency of the references made by interviewees suggests that in their perception, social benefits predominate over social costs, ES-unrelated benefits predominate ES-related benefits and ES-related costs slightly predominate ES-unrelated costs (see Figure 8).

##### ES-Related Social Benefits

**Figure 9:**
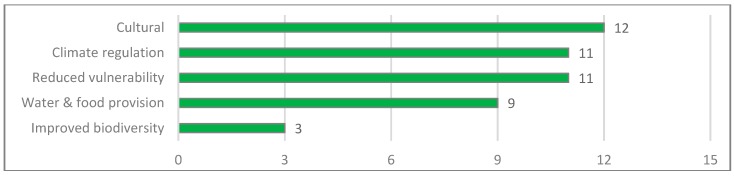
References to ES-related social benefits in the Cartagena case study (own representation).

The most frequently mentioned ES-related benefit refers to culture (see Figure 9). Cultural benefits arose through the recovery of public spaces, which can be used for recreational activities such as sports or bird watching. Some of the interviewees from allied institutions further highlighted that by participating in the project, community members started to rethink their relationship with the surrounding environment and to appreciate nature. This change of mind-set is well illustrated by one of the environmental organizations’ leaders, who said “Before, we were living with our back to the water and now we are turning towards it and view it as an opportunity” (Resident Cartagena, personal communication, 8 February 2018).

The most frequently referenced social benefit in the cluster climate regulation is the provision of shade. Besides the shade, people acknowledged that by capturing carbon dioxide and decreasing the temperature, trees directly contribute to climate regulation and to better air quality (Residents Cartagena, personal communication, 14 March 2018).

Perceived as very important by many interviewees is the reduced vulnerability of UCG6 vis-à-vis climate change. People appreciated the planted mangroves as “natural barriers against floods” (Resident Cartagena, personal communication, 14 March 2018). Adding to that, the mangroves also contribute to limiting illegal settlement as the “reforestation component of the project has helped to build a natural barrier for the illegal invaders of the Ciénaga territory” (Resident Cartagena, personal communication, 6 March 2018). Other interviewees also emphasized the improved waste management in the communities.

Several interviewees considered enhanced food provision and improved biodiversity as an important social benefit. Respondents from the UCG6 stressed the provision of fruits by planted fruit trees, and increased fish stock through the improved water quality (Residents Cartagena, personal communication, 6 March 2018).

##### ES-Unrelated Social Benefits

**Figure 10:**
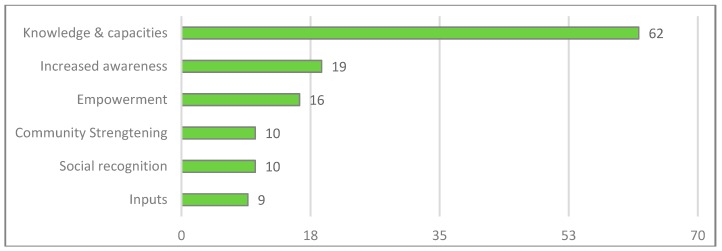
References to ES-unrelated social benefits in the Cartagena case study (own representation).

The most frequently mentioned benefit in the interviews refers to knowledge and capacity-development (see Figure 10). Community members often considered the mere participation in workshops on environmental and organizational topics as beneficial. Implementers and allied institutions emphasized the combination of theory and practice, especially instructions for “do it yourself”-activities that can later on be replicated in the communities (Resident Cartagena, personal communication, 14 March 2018). Community members also highlighted the importance of exchanging experiences, both knowledge transfers at intra-community level (e.g., by organizing own workshops and intergenerational exchanges), and at inter-community level (with people from other neighborhoods or participants at other GIZ project sites). Additionally, the community members attached great value to the involvement and education of the youth, especially through the “environmental guardians” groups and in newly installed tree nurseries in schools (Resident Cartagena, personal communication, 8 March 2018). 

Closely related with knowledge and capacities, our interviews emphasized the increased awareness among community members of climate change impacts and the importance of the ecosystem and ES, for example the importance of mangroves and clean canals for their well-being (Resident Cartagena, personal communication, 8 March 2018). 

The project empowered local organizations as well as individual community members, for instance through participation in a diploma course on climate change and EbA, which “helped to raise [their] self-esteem, because they felt valued and respected” (ministry staff Bogotá, personal communication, 6 February 2018). Community members also highlighted the empowerment of children, as they “now behave like future leaders” (Resident Cartagena, personal communication, 8 March 2018).

Another important benefit brought up by our interviewees is the strengthening of communities, especially by promoting the creation of community organizations, by offering cultural activities for children and by bringing different local stakeholders such as educational institutions, environmental organizations and the private sector together (Resident Cartagena, personal communication, 15 March 2018). Some interviewees emphasized that the project strengthened the cohesion between the different communities of UCG6, also through exchanging experiences among each other and building networks with other organizations and neighborhoods (Resident Cartagena, personal communication, 13 March 2018).

Social recognition as an ES-unrelated social benefit was mainly mentioned by members of the community, not by implementers. A representative of a local environmental organization stated that participating in the project leads to “recognition of the organization and its work inside and outside the neighborhood” (Resident Cartagena, personal communication, 8 March 2018). An adolescent interviewee, who was participating in the environmental guardians’ activities, mentioned that even criminal gangs respect the activities of the group, because they understand that they are “working for everybody’s wellbeing” (Resident Cartagena, personal communication, 14 March 2018). A reason for the importance community members attach to social recognition is that UCG6 has the image of a problematic area. 

Material benefits in the form of inputs included, among others, donation of plants, trees and seedlings, but also working tools and equipment (e.g., rakes, shovels, and machetes).

##### ES-Related and ES-Unrelated Social Costs

To a far lesser extent, the interviewees referred to social costs that arise in the context of the project implementation. 

While the implementing organization and the national and local project partners did not address any social costs at all, the case was different for community members. The ES-related social cost most prominently referred to is social tension, arising due to trade-offs and different usage of the ecosystems. While protection and reforestation of certain areas improve the health of ecosystems, a protected area might also prevent other groups from using the land for settling and cutting down mangroves for construction purposes (Resident Cartagena, personal communication, 8 March 2018). While these are illegal activities, those engaged in them often have little choice. During one of the field visits, a group of adolescents was observed hunting doves and lizards. They were then immediately told to leave, as it is now forbidden to hunt on the premises of the protected area (Resident Cartagena, personal communication, 14 March 2018).

Interviewees pointed out, that environmental measures are sometimes not compatible with safety concerns. Planted trees can interfere with electricity cables or when reforested areas serve as hiding places for thieves or drug addicts (Residents Cartagena, personal communication, 6 March 2018).

ES-unrelated social tensions refer to conflicts. Conflicts occur, for example, between established community members and new comers that “don’t give the environment the space that is needed” (Resident Cartagena, personal communication, 8 March 2018).

Finally, interviewees addressed unsatisfied expectations that emerged from project implementation. Due to irresponsible behavior of individuals, e.g., people stealing trees, and careless maintenance, e.g., when local authorities accidentally destroyed some of the planted trees when cleaning canals’ sediments, potential benefits never materialized (Resident Cartagena, personal communication, 14 March 2018).

#### 4.2.2. Decisive Factors

##### Agent-Level Factors

Human capital of actors clearly influenced the generation of benefits and costs. For Cartagena, knowledge about the effects of climate change, increased heat or sudden climate events such as rains, made project participants more likely to acknowledge that their actual participation can generate ES-related benefits to them and the community overall, whereas lack of awareness constitutes a restricting factor for the effectiveness of the EbA project: cleaning the canals to prevent flooding will not prove to be fruitful if waste disposal in the canals continues due to a lack of awareness.

Social capital has positively influenced the generation and distribution of benefits. Cooperating with actors that are well known and well connected within the community contributes to the performance of the EbA project and vice versa. In the project area, GIZ is cooperating with local environmental organizations whose leaders are familiar with the communal structure and the present socio-ecological challenges. Invasive settlers, i.e. migrants who illegally occupy free spaces in the UCG6 and surrounding districts to construct shelters (mainly near canals and in high-risk areas), are excluded from any activity organized by the local environmental organizations, because the organizations do not want to legitimize illegal settlement that harms the environment and community structure. The exclusion of informal settlers from environmental activities and capacity buildings is problematic because it enhances the social divide between “established” members of the communities and the “illegals”, and because it marginalizes the latter in getting any project benefits.

Moreover, physical capital helps to generate and, more importantly, to distribute social benefits (e.g., awareness raising, capacity building, etc.). One of the local environmental organizations owns a property near the Ciénaga, which is used to conserve nature, to reforest mangroves but also to organize excursions and workshops for community members (Residents Cartagena, personal communication, 14 March 2018).

The community members’ preferences and attitudes towards the EbA project and stakeholders involved (i.e., implementing organizations) are important determinants for how the people actually engage in the project. An implementer stated that one of their strengths was their ability to convince people of the project measures: “We had a good relationship with the stakeholders and they were interested in what we were doing. It helped a lot to involve them in the different steps of implementation, so they knew we would not only call them in the beginning but that they were really part of capacity development and the coordination of measures” (Implementer Cartagena, personal communication, 6 February 2018). 

People who showed interest and committed themselves to the project from the beginning were more likely to receive benefits individually because they actively participated in the project measures. A general interest in, and commitment to, community activities is often a prerequisite for both the generation and the distribution of social benefits. In the context of educational activities, only children whose parents are involved in communal work or are long-time established can participate in these activities. This is problematic because it excludes migrant children from participating (Resident Cartagena, personal communication, 10 March 2018). 

According to members of the local environmental organizations, ownership plays a central role for the sustainability of EbA projects: “We are also empowering the communities. [...] We gave them a tree to plant and said, ‘Now this is your tree’. This gives them a feeling of responsibility and ownership and people really take care of their tree” (Resident Cartagena, personal communication, 8 March 2018).

It is important to consider that in Cartagena, project beneficiaries do not directly depend on the Ciénaga for their livelihood: “It is mainly an urban population and therefore they don’t know the benefits of the ecosystems. However, in this area, people depend directly on healthy mangroves” (Implementer Cartagena, personal communication, 14 March 2018). This aspect affects the way participants perceive EbA projects and value their impacts: “The measures and activities have to be adapted to the circumstances of an urban environment. We have to work with these people and sensitize them somehow” (Implementer Cartagena, personal communication, 14 March 2018).

##### Structural Factors

Stakeholder participation and communication are important—between all stakeholders involved and at all stages of a project (i.e., planning, implementation and evaluation). Different approaches were developed with key stakeholders, including the local communities and neighborhoods. In the words of one implementer, “making friends at all levels—within the communities, at local, regional and national level” is considered important for effective project implementation (Implementer Cartagena, personal communication, 6 February 2018). By partnering with a local organization running social and environmental activities (e.g., City of Cartagena and Fundación Social) in the UCG6 the alignment and cooperation of all actors created a certain level of trust within the community of the UCG6.

With regard to institutional context, environmental law enforcement in Cartagena and in the UCG6 is weak: “The state is almost absent and environmental law enforcement is non-existent on this site” (Resident Cartagena, personal communication, 7 March 2018). Land filling for housing and real state are not controlled by the authorities in Cartagena. This results in the decline of the Ciénaga and other ecosystems. Environmental issues are only addressed by the local self-organized organizations that receive scant support from public authorities (Resident Cartagena, personal communication, 8 March 2018). Weak law enforcement not only hinders the generation of social benefits for the most vulnerable, but also severely impedes upon the sustainability of EbA-related activities.

With regard to the local context, implementers focused on local knowledge, “working with universities and schools, promoting research to solve the local problems [...]. The goal was to have a process that brings together students, NGOs and communities in order to produce research that is really informed by local stakeholders.” (Resident Cartagena, personal communication, 7 March 2018). Implementers appear to be aware of the importance of using existing local knowledge: “Local knowledge is very valuable; the involvement of local actors is key” (Implementer Cartagena, personal communication, 6 February 2018).

The implementer sought to build on existing practices and networks (e.g., Fundación Social and the local environmental organizations) and adapted capacity building measures that fostered interaction. Using pre-existing community organizations accelerated the implementation procedures and enabled the use of existing synergies: “GIZ provided its expertise on EbA, while Fundación Social provided the networks and contacts of the social leaders in the area” (Resident Cartagena, personal communication, 6 March 2018). 

## 5. Discussion

The wide range of identified categories stems from our perceptions-based research approach. The majority of social benefits and costs encountered in the data were perceived as the result of project measures by the local communities. The research results indicate that the social dimension of EbA projects is two-fold: not only are social outcomes part of it (e.g., social cohesion or people’s behavior towards nature and towards each other), but also various ES-related impacts. The latter include the different ES as categorized in the Millennium Ecosystem Assessment (MEA) framework. Our interviewees not only regarded the improvement of the more apparent ES as social benefits (i.e., provisioning services and cultural services), but also referred to the more “invisible” benefits such as regulating services (e.g., climate regulation).

Moreover, community members in both case studies perceived ES-unrelated social benefits that had not been anticipated or envisaged by the implementing organizations. The collective work on environmental topics appears to be a tool for effective community strengthening by promoting social cohesion and contributing to conflict resolution. It may lead to individual as well as group empowerment. The perceived outward social recognition seems to benefit from EbA projects as well, e.g., the improved image of UCG6. 

The emerging picture of a very positive perception of the EbA project in Cartagena opposed to the many ES-unrelated social costs in Chingaza has to be put into context. It is particularly important to consider the temporal dimension. While INAP ended in 2011, project implementation in Cartagena was still ongoing by the time we conducted the research. People’s perceptions regarding INAP might have changed in the course of time, also influenced by the fact that some of the activities were not maintained after project implementation. At the time, participants of the Cartagena project were interviewed, who could not yet look back at the project from a distance.

EbA projects are developed in, and are influenced by, a dynamic socioeconomic context. In the general discourse, communities involved in EbA projects are labeled as being better adapted and less vulnerable to the risks of climate change. It is questionable to what extent this is true, as the causal relationship between the project measures and reduced vulnerability is hard to prove. In the case of Cartagena, this means, for instance, that the regular cleaning of the canals in UCG6 does not automatically lead to a reduced incidence of flooding because other variables might also have an impact in this case. As our research team consisted of social scientists and focused on the social dimension of EbA projects, it is not within the scope of our research to come to a conclusion on the “real” state of the communities’ adaptation. However, in the light of the relatively low volume of funds committed to the projects their duration, and lack of monitoring, we questioned the projects’ potential to adapt communities to climate change. The ambitious goal of EbA to adapt whole communities may lead to unsatisfied expectations by community members concerning their realistic capacities. Implementers, on the other hand, have difficulties measuring the reduced level of vulnerability as a direct outcome of their respective project. This could consequently complicate the justification of EbA projects in general. A more cautious handling of promises concerning the reduced vulnerability after the conclusion of EbA projects could lead to more realistic expectations. Therefore, targets of EbA projects should leave some room for adjustment in response to the evolution of climate change impacts. 

Until now, many EbA projects remain pilot projects—as was the case in the two projects analyzed in Colombia. Between 2006 and 2011, INAP was the first adaptation project in Colombia. In 2014, the EbA project in Cartagena was officially labeled a pilot project [45]. Given the number of pilots remaining worldwide, questions regarding the necessity of upscaling these pilots could be raised.

As the results of our two case studies demonstrate, social benefits exceed social costs in the process of EbA project implementation. If further evidence for the effectiveness of EbA projects and their contribution to an improved adaptation of local communities is provided in this context, an upscaling of EbA—vertically into policies and horizontally into regions or practices—could be a promising path. 

When examining the EbA projects within its local context, some important reflections can be drawn from the perspective of political ecology. The communities of both case studies actively engaged in adapting themselves to the negative effects of climate change. However, their adaptive capacities and thus the success of their actions were not only threatened by the actions of more powerful actors in their surroundings. In the case of Cartagena, the government and the tourism industry were developing infrastructure (e.g., new buildings and roads) in the immediate vicinity of the Ciénaga. By cutting down nearby mangroves and dumping wastewater into the swamp, real estate and industry contributed to the destruction of the ecosystem that local communities were trying to restore with their activities. Furthermore, the livelihoods of already underprivileged people were impaired by new restrictions concerning their settlement and hunting customs. The situation was similar in Chingaza, where large-scale cattle ranchers polluted the rivers and engaged in deforestation upstream. Instead of targeting those large-scale farmers, INAP addressed small-scale landowners in the local communities, which led to an unequal distribution of responsibilities and burdens on the local level. The same can be said about the protection of the water storage capacity in the páramo; the necessity is felt to a higher degree by authorities in Bogotá than in the local communities, but the latter were the ones bearing the burden of environmental protection.

## 6. Conclusions

Our findings indicate that social benefits and costs are multifaceted. In both cases, our interview partners emphasized increased knowledge and awareness of climate change among local communities, gender empowerment, employment opportunities and perceived climate regulation (e.g., shade provision, regulation of floods, wind protection). Reported costs included creation of social tensions, unfulfilled project expectations and the risks of being labeled as “adapted”. Particular benefits and costs also related to the setting where projects were implemented. For example, in the rural setting benefits related to water provisioning, whereas in the peri-urban setting, referred to changing social stigmatization or the creation of public spaces. Costs in the rural setting included, among others, non-recognized land opportunity costs, whereas in the urban setting referred to the creation of hide-outs for criminals. 

While carrying out research, community members also highlighted the contradictions in which EbA projects operate. Munang and Andrews [46] explained that the success of EbA depends largely on involving the local community in the planning and implementation process, while bearing in mind the overall political context and related conflicts. Although our research highlights the importance of the first part of their claim, we found evidence to challenge the second part of it. Many EbA projects are implemented in local communities on a relatively small or pilot scale. However, near these communities, powerful economic actors (i.e., large-scale cattle ranchers and real estate developers) pursue activities that frequently reduce the adaptive capacities of communities, for example by draining wetlands or cutting down trees, which increases the risk of floods. In addition, powerful economic actors tend to circumvent environmental protection laws with the use of political power, usually carrying on with destructive practices towards ecosystems. In this sense, the success of EbA not only depends on the efforts made by the communities themselves but also on environmental law enforcement and on the degree of responsibility that powerful economic agents are willing to adopt for these shared-ecosystems.
